# Giant pituitary adenomas: an institutional experience with 289 surgically treated patients

**DOI:** 10.1007/s00701-026-06830-6

**Published:** 2026-03-25

**Authors:** Victoria Antonia Binder, Yining Zhao, Julia Sandra Breu, Moritz Repschläger, Rudolf Fahlbusch, Michael Buchfelder

**Affiliations:** Department of Neurosurgery, University Hospital of Erlangen, Erlangen, Germany

**Keywords:** Giant pituitary adenomas, Transsphenoidal pituitary surgery, Transcranial pituitary surgery, Surgical outcome, Hypopituitarism

## Abstract

**Background:**

Giant pituitary adenomas (GPA) are considered difficult to treat and the operative procedures are associated with more complications. This study aimed to assess treatment strategies of GPAs in a large consecutive and uniformly documented series in a single specialized center.

**Methods:**

A total of 289 patients with GPA who underwent primary surgery in our department between December 1982 and December 2022 were analyzed in this retrospective study. GPAs were defined by a maximum diameter of ≥ 4 cm in at least one plane. Patients were reviewed for endocrine, radiological and ophthalmological outcomes as well as complication and mortality rates.

**Results:**

The mean maximum tumor diameter was 4.6 ± 0.7 cm. 201 patients (69.6%) underwent transsphenoidal and 36 patients (12.4%) underwent transcranial surgery only. 52 patients (18.0%) underwent a combined approach within a few weeks. Gross-total resection (GTR) was achieved in one-fifth (*n* = 52) of the patients. It was dependent, among other factors, on patients’ tumor size and tumor extension. Severe complications such as tumor apoplexy, meningitis or cerebrospinal fluid leaks occurred in 5.9%, 3.5% and 2.8% of patients, respectively. Seven deaths (2.4%) occurred in the early postoperative period. The median follow-up time was 76 months, at which point 70.2% of patients showed a stable condition without requiring further treatment.

**Conclusion:**

Generally, the treatment of giant pituitary adenomas remains a significant challenge. Although the transsphenoidal approach achieved good results, tumor size and configuration often required a transcranial approach or a combination of different approaches. It is important to consider individual patient and tumor characteristics when selecting the most appropriate surgical approach.

## Introduction

Large and invasive pituitary adenomas are considered particular challenges for the neurosurgeon. When Symon et al. [65, 66] compared their outcomes after craniotomy for adenomas with suprasellar extension, they found a clear difference between the moderately sized and very large ones in terms of complications and outcomes and consequently coined the term “giant adenomas” for those, which measured more than 40 mm at least in one plane. Thereafter, the evolution of computerized tomography (CT) and magnetic resonance (MR) imaging allowed the precise depiction of a pituitary tumor at presentation and quantification of the extent of resection. It became the general experience that size is a major determinant of remission in patients with acromegaly, prolactinomas and Cushing’s disease [7, 19, 53]. A variety of publications reported on the institutional outcomes of surgical treatment of giant pituitary adenomas or PitNETs, respectively, according to the most recent update of the WHO classification of pituitary tumors [10]. Some applied microsurgical [24, 25, 36, 50, 51, 63, 75], other pre-dominantly endoscopic techniques [11, 15, 17, 26, 41, 44, 45, 68, 70]. The presence or absence of invasion into the cavernous or sphenoid sinus, clivus and arachnoid are other factors, apart from mere size, which determine the amount of resectablity together with the configuration of a tumor [46], its consistency and the condition of the patient. The cooperation with endocrinologists, radiation oncologists and pathologists in an expert center allows to utilize personalized multimodal treatments [32]. We considered it worthwhile to report our extensive institutional experience with giant pituitary adenomas treated in a dedicated center. They were treated with standardized surgical protocols by two surgeons (MB and RF), in which the size, configuration and extent defined whether a transsphenoidal or transcranial approach was utilized and, if both were applied within a few weeks, which sequence was used. Preoperative medical treatment was mainly performed in prolactinomas and in a few patients with acromegaly at the discretion of the referring physician. In contrast to many other reports, this is a consecutive series of 289 primary managements of giant pituitary adenomas out of a total of 6172 patients operated in this single center within a 40-year period between 1982 and 2022.

## Material and methods

This study included 289 consecutive patients who underwent primary surgery for giant pituitary adenoma at the Department of Neurosurgery, University Hospital of Erlangen between December 1982 and December 2022. Inclusion criteria were a maximum tumor diameter ≥ 4 cm on preoperative imaging. The patients' data regarding pre- and postoperative clinical features, as well as endocrine, radiological, and ophthalmological outcomes, complication rates, and mortality rates, were analyzed retrospectively.

Endocrinological assessment was performed using commercially available test kits. Basal levels of prolactin (PRL), growth hormone (GH), luteotropin (LH), follicle-stimulating hormone (FSH), testosterone in men and estradiol in women, cortisol, thyroid-stimulating hormone (TSH), free triiodothyronine (fT3) and free thyroxine (fT4) were assayed for each patient. Reliable measurements of IGF-1 have only been possible since 2013.

Radiological imaging was performed preoperatively and about 3 months after the operation. CT preceded 35 operations (10.2%) and MRI 297 operations (86.3%), while both modalities were used before another 12 procedures (3.5%). A distinction was made between gross-total resection (no radiological evidence of tumor remnants) and incomplete resection. Intraoperative imaging was introduced in our department in 1996 [20]. Since then, it has been used in 75 operations (22.0%), 71 of which were transsphenoidal only.

The pre- and postoperative ophthalmological examination included visual acuity and visual field defects. The assessment was made using a modified score according to the guidelines of the German Ophthalmological Society (GOS) [33].

Patients underwent either transsphenoidal or transcranial surgery alone or a two-staged strategy. The surgical procedures of the transsphenoidal and transcranial approach used in our department have been published before [5–7]. The two-staged strategy usually involved a combination of transsphenoidal and transcranial surgery, with the second surgery performed within six months of the first.

Complications occurring within 30 days of surgery were considered. They included death, tumor apoplexy, meningitis, CSF-leaks, cranial nerve (CN) palsies, loss of vision, postoperative hypopituitarism, transient and permanent diabetes insipidus (DI) and hyponatremia.

Patient data were analyzed using Microsoft Excel (version 16.77) and IBM SPSS Statistics (version 29.0.1.0). The following statistical tests were used: Mann–Whitney-U test, chi-square test and Fisher’s exact test. A p < 0.05 was considered significant and all p-values were two-sided.

## Results

### Patient and tumor characteristics

**Fig. 1:**
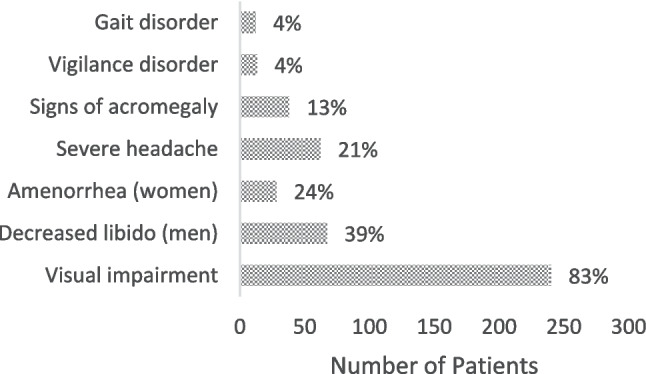
Preoperative symptoms of the 289 patients with GPA

Out of a total of 289 patients, 174 were male patients (60.2%) and 115 were female patients (39.8%). Age ranged from 11 to 85 years, with a mean age of 51.8 years (± 16.4 years). Most patients (83%, *n* = 240) complained of visual deterioration. Diplopia was reported to have been experienced by 25 patients (8.7%), 17 of whom patients had manifest CN III, IV or VI palsies. Table 5 gives an overview of CN palsies preoperatively. Severe headache was noted in 62 patients (21.5%) and 38 patients had signs of acromegaly (13.1%), such as an enlargement of the hands, feet or face. Preoperative hypopituitarism of one or more axes was present in 194 patients (67.1%). A diminished libido was reported in 67 patients (23.1%), and amenorrhea was noted in 28 of the female patients (24.3%). Qualitative or quantitative disturbances of consciousness were present in 17 patients (5.9%), a further 12 patients (4.2%) were found to have gait disorders. Figure 1 displays the most common preoperative symptoms.

The mean tumor diameter was 4.6 cm (± 0.7 cm; range 4–9 cm) with a mean tumor volume of 33.5 cm^3^ (± 23.2 cm^3^; range 4 cm^3^–180 cm^3^). There were 194 patients (67.1%) with non-functioning adenomas, 44 patients (15.2%) with prolactinomas, 43 patients (14.9%) with acromegaly, 3 patients (1.0%) with Cushing's disease, 3 patients (1.0%) with gonadotropin-secreting adenomas and 2 patients (0.7%) with thyrotropin-secreting adenomas.

A suprasellar extension of the tumor was present in 236 patients (81.7%) and almost as many tumors (228 patients, 78.9%) had a parasellar extension. Sphenoidal sinus extension, retrosellar extension, temporal extension and subfrontal extension were found in 152 patients (52.6%), 74 patients (25.6%), 49 patients (17.0%) and 18 patients (6.2%), respectively. Hydrocephalus due to obstruction of the foramen of Monroi was present in 32 patients (11.1%). Based on the surgeon's impression, radiological signs of invasive growth, and/or histologically detected adenoma cells in sphenoid sinus or bone biopsies, a total of 253 adenomas (87.5%) showed invasive growth. Table 1 shows the baseline tumor characteristics.

**Table 1:** Baseline tumor characteristics of the 289 patients with GPA

Tumor characteristics	no.	%
Maximum tumor diameter (cm)	4.6 ± 0.7 (range 4–9)	
Maximum tumor volume (cm^3^)	33.5 ± 23.2 (range 4–179.6)	
Functional classification		
- Non-functioning adenoma	194	67.1
- Prolactinoma	44	15.2
- GH-secreting adenoma	43	14.9
- ACTH-secreting adenoma	3	1.0
- Gonadotropin-secreting adenoma	3	1.0
- TSH-secreting adenoma	2	0.7
Suprasellar extension with optic nerve compression	236	81.7
Parasellar extension	228	78.9
Sphenoidal sinus extension	152	52.6
Retrosellar extension	74	25.6
Temporal extension	49	17.0
Subfrontal extension	18	6.2
Hydrocephalus	32	11.1
Invasiveness	253	87.5

### Surgical management and outcome

A total of 344 operations were performed on the 289 patients, including a variety of two- and three-staged procedures. For most patients (*n* = 201, 69.6%) a single microscopic transsphenoidal approach was our first choice, often endoscopy-assisted. Single transcranial surgery was performed in 36 patients (12.5%). A total of 49 patients (16.9%) underwent a two-staged procedure, while 3 patients (1.0%) underwent up to 3 operations within 6 months. In our two-staged treatment strategy, adenomas were usually first removed via a transsphenoidal approach, followed by a transcranial surgery (34 patients, 11.8%). Intraoperative MR-imaging was used in 75 operations (out of 341 operations, 22.0%), 71 of which were transsphenoidal. Thirteen patients (4.5%) received a shunt prior to undergoing surgery. Table 2 provides an overview of the surgical approaches.

**Table 2:** Surgical approaches in the patients treated for GPA

Surgical Approach	no.	%
Only TS	201	69.6
Only TC	36	12.5
TS + TC	34	11.8
TC + TS	10	3.4
TS + TS	3	1.0
TC + TC	2	0.7
Three operations	3	1.0
Preoperative Shunt	13	4.5
Intraoperative MRI (n = 341)	75	22.0

GTR was achieved in 52 patients (20.5%). Subtotal and partial resection was noted in 159 patients (62.6%) and 43 patients (16.9%), respectively. Table 3 shows the association between the extent of resection and patient and tumor characteristics. There was no significant correlation with age, sex, the endocrinological tumor type or parasellar and sphenoid sinus extension. Incomplete tumor resection was associated with a larger tumor diameter and volume compared to GTR, which had significantly smaller tumors in diameter and volume (4.7 cm vs. 4.4 cm, *p* = 0.005 and 34.9 cm^3^ vs. 28.1 cm^3^, *p* = 0.032).

**Table 3:** Comparison of patient and tumor characteristics in relation to GTR or incomplete tumor resection

	GTR *n* = 52	Incomplete resection *n* = 202	***p***-value
Patient characteristics			
Age (yr)	53.2 ± 14.7	50.0 ± 16.3	ns
Men, no.	35 (67.3%)	119 (58.9%)	ns
Tumor characteristics			
Non-functioning adenomas, no.	39 (75.0%)	127 (62.9%)	ns
Maximum tumor diameter (cm)	4.4 ± 0.4	4.7 ± 0.8	0.005
Maximum tumor volume (cm^3^)	28.1 ± 15.5	34.9 ± 25.1	0.032
Parasellar extension, no.	38 (73.1%)	167 (82.7%)	ns
Retrosellar extension, no.	6 (11.5%)	61 (30.2%)	0.006
Sphenoid sinus extension, no.	30 (57.7%)	106 (52.5%)	ns

An extension to retrosellar areas was also connected to a higher likelihood of incomplete resection (*p* = 0.006). GTR was possible in 6 out of 67 patients (9.0%) with preoperative retrosellar extension, compared to 46 out of 187 patients (24.6%) without retrosellar extension. Although parasellar extension showed no significant influence on incomplete tumor resection there was a trend towards it. While GTR was achieved in only 38 out of 205 patients (18.5%) with preoperative parasellar extension, it was achieved in 14 out of 49 patients (28.6%) without preoperative parasellar extension. A total of 116 patients (40.1%) obtained postoperative radiotherapy.

A reoperation was required for 38 patients: In 13 patients due to progression of a residual tumor, in 13 patients due to a symptomatic residual tumor, and in 12 patients for recurrence. For the purposes of this study, only tumors that were deemed to be completely removed by imaging after primary treatment were considered recurrent. This corresponded to a recurrence rate of 12 out of 52 (23.1%) completely resected adenomas. The median follow-up time was 76 months, at which point 70.2% of patients showed a stable condition without requiring further treatment.

**Fig. 2:**
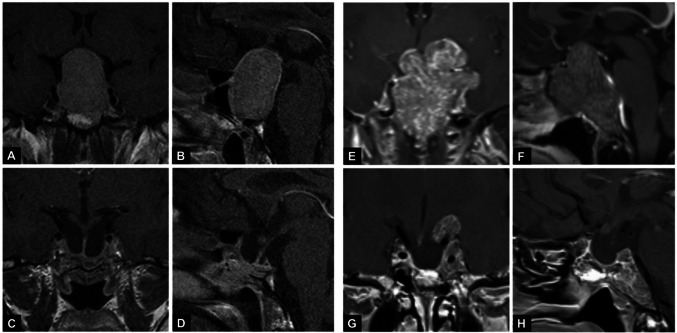
T1-weighted MRI in coronal (**A**, **C**, **E**, **G**) and sagittal (**B**, **D**, **F**, **H**) planes of a 39-year-old patient before (**A**, **B**) and after (**C**, **D**) a single transsphenoidal operation with GTR and a 48-year-old patient before (**E**, **F**) and after (**G**, **H**) a combined approach (TS + TK) with incomplete tumor resection. Both patients suffered from non-functioning pituitary adenomas and presented with visual compromise, which recovered completely after surgery

Figure 2 shows examples of complete and incomplete tumor resection, as demonstrated by the preoperative and postoperative MRIs.

### Endocrinological outcome

#### Prolactinoma

All patients suspected of having a prolactinoma were treated with dopamine antagonists. As a result, 67 patients (23.2%) received treatment, including those with prolactinoma (44 patients, 15.2%) and those with non-tumorous hyperprolactinemia (23 patients, 8.0%). Prolactin levels could be significantly reduced in patients with prolactinomas (*p* = 0.003). However, endocrinological remission was present in only 21.1% (*n* = 38) of these patients about three months after surgery.

#### Acromegaly

Of 43 patients with acromegaly, 34 (79.1%) were treated with somatostatin analogues. No patient showed suppression of hGH to below 1 ng/ml during the oGTT prior to surgery. About three months after the surgical treatment hGH could be suppressed in 6 patients (24.0%, *n* = 32). Since IGF-1 levels could be reliably assessed, 7 patients with acromegaly were managed surgically, of whom 1 patient (14.3%) achieved postoperative endocrine remission.

#### Cushing’s disease

In all three patients with Cushing's disease, the dexamethasone suppression test could not achieve suppression to below 2 µg/dl, neither pre- nor postoperatively. The same applied to ACTH levels which did not show a significant decrease. In addition, one of the tumors was later found to convert to pituitary carcinoma.

### Visual outcome

Pre- and postoperative data about ophthalmological outcome were available for 239 patients. Improved vision was observed in 141 patients (59.0%). The vision of 47 patients (19.7%) deteriorated, while it remained unchanged in 51 patients (21.3%). According to the GOS guidelines, a mean preoperative visual impairment score of 35.2 (± 29.3) was determined. The mean postoperative score was 28.4 (± 30.3), which was significantly lower than the preoperative score (*p* < 0.001). Out of 25 patients reporting diplopia preoperatively, 16 patients (64.0%) described an improvement after surgical treatment.

### Complications

**Fig. 3:**
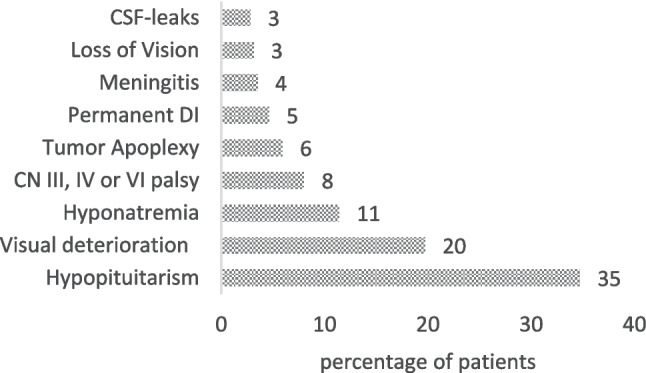
The most frequent complications following surgical treatment for GPA

Among the 4 most frequent surgical approaches, the trans-cranial approach had the highest complication rate (*n* = 25, 69.4%). The lowest complication rate occurred in the transsphenoidal-transcranial cohort (*n* = 2 out of 6 surgeries, 33.3%; *p* = 0.002). Figure 3 illustrates the most frequent complications, while Table 4 shows an overview of the complications in relation to the surgical approach. We restricted the analysis to univariable comparisons, given the low numbers of events for each complication and the presence of small subgroup sizes.

**Table 4:** Complications of surgical treatment in relation to the sequence of operative approaches

Surgical Approach	Total *n* = 289	TS *n* = 201	TC *n* = 36	TS + TC *n* = 34	TC + TS *n* = 10	*p*-value
Mortality	7 (2.4%)	2 (1.0%)	3 (8.3%)	1 (2.9%)	1 (10.0%)	0.016
Postoperative hemorrhage	17 (5.9%)	12 (6.0%)	4 (11.1%)	1 (2.9%)	0	ns
Meningitis	10 (3.5%)	8 (4.0%)	0	1 (2.9%)	1 (10.0%)	ns
CSF-leak^a^	8 (2.8%)	3 (1.5%)	1 (2.8%)	3 (8,8%)	0	ns
Amaurosis	9 (3.1%)	3 (1.5%)	4 (11.1%)	1 (2.9%)	1 (10.0%)	0.014
CN III, IV or VI palsy^b^	23 (8.0%)	8 (4.0%)	8 (22.2%)	4 (11.8%)	2 (20.0%)	< 0.001
Hyponatremia	33 (11.4%)	29 (14.4%)	2 (5.6%)	2 (5.9%)	0	ns
Hypopituitarism^c^	33/95 (34.7%)	15/58 (25.9%)	8/15 (53.3%)	7/15 (46.7%)	2/5 (40.0%)	ns
Transient DI	14/281 (5.0%)	10/199 (5.0%)	3/33 (9.1%)	0/33	1/8 (12.5%)	ns
Permanent DI	13/281 (4.6%)	9/199 (4.5%)	2/33 (6.1%)	2/33 (6.1%)	0/8	ns

^a^One additional patient who underwent a three staged procedure

^b^One additional patient who underwent a TC + TC procedure

^c^One additional patient out of 2 patients who underwent a three staged procedure

Surgical mortality was 2.4% (7 patients). Three deaths occurred within 30 days and 4 deaths within 60 days after surgery. These patients were treated as follows: Two patients underwent transsphenoidal surgery, 3 patients underwent transcranial surgery only and 2 patients underwent a combined approach (*p* = 0.016). Four out of those 7 patients died from postoperative tumor apoplexy with hemorrhagic hydrocephalus. Implantation of an external ventricular drainage could not prevent death in these patients. One patient deceased from intracerebral hemorrhage, herpes simplex virus type 1 encephalitis and multiple pulmonary embolisms. Another patient developed generalized convulsive status epilepticus secondary to a chronic subdural hematoma and eventually died from metabolic disorders. Sudden cardiorespiratory arrest resulted in death of one patient, the exact etiology remaining unclear.

Postoperative hemorrhage occurred in 17 patients (5.9%). Of these patients, 12 had previously undergone transsphenoidal surgery, 4 had undergone transcranial surgery and 1 had initially undergone transsphenoidal surgery, followed by transcranial surgery (hemorrhage after the first operation) (*p* = 0.492). Five patients underwent surgical evacuation of the hematoma, 7 required external ventricular drainage, and 4 underwent ventriculo-peritoneal shunt implantation.

Ten patients (3.5%) developed postoperative meningitis. Of these, 8 developed it after isolated transsphenoidal surgery, while 2 patients underwent two-staged surgery (1 patient transsphenoidal-transcranial and 1 patient transcranial-transsphenoidal), with meningitis developing after the second operation (*p* = 0.379).

CSF-leaks resulting from the operation occurred in 8 patients (2.8%). Of the patients undergoing transsphenoidal surgery, 3 developed CSF-leaks, as did 1 of those undergoing transcranial surgery and 3 of those undergoing combined transsphenoidal-transcranial surgery (*p* = 0.086). One patient undergoing a three-staged surgical procedure also developed a CSF leak. In the combined procedures, CSF leaks were observed after 2 transsphenoidal and 2 transcranial operations. A total of 9 patients (3.1%) experienced either unilateral or bilateral loss of vision following surgery. Of these, 2 of those who underwent transsphenoidal surgery, 3 of those who underwent transcranial surgery, and 1 each of those who underwent transsphenoidal-transcranial surgery and transcranial-transsphenoidal surgery experienced unilateral amaurosis. Bilateral amaurosis affected 1 transsphenoidally operated and 1 transcranially operated patient (*p* = 0.014).

Manifest cranial nerve III, IV or VI palsy occurred in 23 patients (8.0%). In the transsphenoidal and transcranial cohort, 8 patients were affected, while in the transsphenoidal-transcranial and transcranial-transsphenoidal cohorts, 4 and 2 patients were identified, respectively (*p* < 0.001). Another patient in the transcranial-transcranial cohort developed an abducens nerve palsy. Table 5 gives an overview of the new nerve palsies of the CN I, II, III, IV and VI postoperatively. No CN V palsy occurred.

**Table 5:** CN I, II, III, IV and VI palsies pre- and postoperatively

	CN I palsy	CN II palsy	CN III palsy	CN IV palsy	CN VI palsy
**Preoperatively** (*n* = 289)^a^	7 (2.4%)	240 (83.0%)	14 (4.8%)	1 (0.3%)	3 (1.0%)
**Postoperatively** (*n* = 289)	1 (0.3%)	47/239 (19.7%)^b^	15 (5.2%)	2 (0.7%)	7 (2.4%)^c^
TS (*n* = 201)	1 (0.5%)	27/170 (15.9%)	4 (2.0%)	0	4 (2.0%)
TC (*n* = 36)^d^	0	8/24 (33.3%)	8 (22.2%)	0	1 (2.8%)
TS + TC (*n* = 34)	0	8/31 (25.8%)	2 (5.9%)	2 (5.9%)	0
TC + TS (*n* = 10)	0	2/7 (28.6%)	1 (10.0%)	0	1 (10.0%)

^a^Regarding CN III, IV and VI palsies, one patient had both a CN III and CN IV palsy preoperatively

^b^Two patients in the TS + TS cohort, 1 patient in the TC + TC cohort and 3 patients in the three-staged procedure cohort complained of visual impairment (CN II palsy)

^c^One additional patient in the TC + TC cohort developed a CN VI palsy

^d^Regarding CN III, IV and VI palsies, one patient in the TC cohort developed both a CN III and a CN VI palsy

The main endocrine complications were postoperative hypopituitarism and hyponatremia. Following surgery, 33 out of 95 patients (34.7%) who were not affected by anterior hypopituitarism prior to surgery developed insufficiency in at least one hormonal axis. The lowest rate was observed in the transsphenoidal cohort (25.9%), but no significant difference was found between the surgical approaches.

Hyponatremia occurred in 33 patients (11.4%). Twenty-nine patients underwent transsphenoidal surgery, 2 underwent transcranial surgery and 2 underwent transsphenoidal-transcranial surgery (*p* = 0.236). It could be controlled in all patients by restricting their fluid intake or by administering tolvaptan (vasopressin antagonist). 

Transient and permanent DI developed in 14 (5.0%) and 13 (4.6%) patients, respectively. Patients who developed permanent diabetes insipidus (DI) were treated as follows: Nine patients underwent transsphenoidal surgery, 2 underwent transcranial surgery, and 2 underwent a transsphenoidal-transcranial procedure (*p* = 0.797). Data were available for 281 patients; one additional patient had already been diagnosed with DI prior to surgery.

## Discussion

Due to their irregular growth and invasiveness, GPAs require individualized treatment strategies. Whilst the majority of pituitary adenomas can be removed using a transsphenoidal approach, which is associated with low morbidity and mortality rates [7], this approach is not generally considered suitable for all giant adenomas. Especially the tumor localization, extent and configuration determine the surgical approach. According to Micko et al. [46], a greater “neck-to-dome area” ratio leads to a lower likelihood of suprasellar tumor descent. Consequently, this increases the risk of hemorrhagic transformation within residual tumor. We emphasize the importance of interdisciplinary management in these tumors [37, 49, 62]. Almost all giant prolactinomas were initially treated with dopamine agonists, and those which responded with an impressive decrease in prolactin levels and tumor shrinkage were continued on medical treatment [1, 9, 49, 62, 69]. In this report, however, we focus on patients of all endocrinological types in whom we saw an indication for surgery.

At our department 70% of patients with GPA underwent a single transsphenoidal approach. This approach was particularly suitable for adenomas with wide communication between intra- and extrasellar parts of the tumor. When a single transsphenoidal approach was not sufficient, a second surgery, usually transcranial surgery, was added. The transcranial approach was mainly used for tumor growth in subfrontal, temporal or retrosellar areas, as well as adenomas with narrow communication between intra- and extrasellar tumor parts [6, 7]. Therefore, it was either a good supplementary approach or primary single surgery (with each approach accounting for 12% of patients).

Other combinations of transsphenoidal and transcranial approaches were performed in 6% of patients. Performing a transsphenoidal approach first offers the chance of achieving sufficient adenoma resection in a single surgery from below, even in irregularly growing tumors. Based on the surgeons’ intraoperative impression and postoperative imaging, the decision to schedule another surgery can be made, allowing time for the patient to recover. This method of scheduling surgeries at different times differs from the approach of other surgeons, who performed transsphenoidal and transcranial procedures simultaneously [2, 43, 58].

GTR was achieved in 20.5% of patients. It is important to consider that operational methods and tools have changed and improved over the 40-year period of observation. Influencing factors on incomplete tumor resection were identified in this series to be tumor size and tumor extension to retrosellar areas. The maximum tumor diameter and the associated tumor volume was significantly lower in tumors with GTR. The same significant influence on GTR was present in retrosellar grown tumors. Whereas literature has been shown that parasellar tumor extension limits GTR [39, 41, 47, 50], this series only suggests this association.

While most GPAs are non-functioning, it is important to distinguish between hormonal active and inactive tumors for treatment purposes [59, 64]. This is particularly relevant for prolactinomas, for which medical therapy with dopamine agonists is the primary treatment [8, 49, 69]. In this series the endocrinological remission rates were 21.1% and 24.0% for patients with prolactinoma and acromegaly, respectively.

It is important to mention that all prolactinomas which were treated surgically, either had an insufficient response to medical therapy and/or severe symptoms resulting from tumor compression. None of the patients with Cushing's disease achieved endocrinological remission. It has been shown in literature that hormonal remission in hormonal active adenomas can be achieved more frequently in smaller tumors than in larger tumors, when comparing surgically treated adenomas [53, 60]. It is particularly challenging to obtain endocrinological remission in giant adenomas. It emphasizes the reasonable distinction between micro-, macro- and giant adenomas [28, 31, 34, 66].

The perioperative mortality was 2.4%, which is comparable to other large series [11, 16, 25, 35, 50, 51, 56, 72, 74]. Table 6 shows an overview of representative literature on surgically treated GPAs [3, 4, 11–14, 17, 18, 21, 23–27, 29, 30, 35, 36, 38, 41, 42, 44, 45, 50–52, 54–57, 63, 66, 68, 70–76]. The most common cause was postoperative tumor apoplexy, with no significant difference observed between the surgical modalities. Of the 17 patients with postoperative hemorrhage, 4 patients died, indicating that survival probability decreases when this complication occurs. The greatest risk of postoperative hemorrhage arises from tumor remnants. In other large series postoperative hemorrhage was also a major cause of death [26, 44, 50, 61]. A statistically significant difference in mortality rates was observed between the surgical modalities, with the transsphenoidal operations resulting in the lowest mortality rate. In the relevant literature, this procedure has been found to be less complication-affected and is therefore preferred [40, 61]. In a recent meta-analysis Tang et al. [67] have compared surgical options. They also found that the transsphenoidal approach in all its fashions was associated with higher rates of GTR and lower mortality and complication rates. However, as they admit themselves, a compilation of literature data suffers from the heterogeneity of data acquisition and management protocols. Notwithstanding the higher complication and mortality rates associated with the transcranial approach, it should not be disregarded. On average, the tumors treated with this modality were larger and anatomically more challenging or inaccessible for transsphenoidal surgery.

**Table 6:** Overview of representative literature on surgically treated GPAs

Reference	Period	Patients (no.)	Surgical Approach	GTR (%)	Mortality (%)	RT (%)	Recurrence (%)
Symon et al. 1979 [66]	1968–1978	16	transcranial	na	18.7	na	na
Pia et al. 1985 [55]	1953–1983	77	microsurgical transsphenoidal, transcranial	na	14.3	29	29
Goel et al. 1996 [24]	1989–1994	30	microsurgical transsphenoidal, transcranial	0	20	73	na
Goel et al. 2004 [25]	1995–2002	118	microsurgical transsphenoidal, transcranial	29.7	2.5	27.9	na
Barzaghi et al. 2007 [3]	1990–2004	54	microsurgical transsphenoidal	na	3.7	na	na
Mortini et al. 2007 [50]	1990–2004	95	microsurgical transsphenoidal, transcranial	14.7	3.2	33.6	15.7
Xue-Fei et al. 2008 [70]	2000–2006	54	endoscopic endonasal, transcranial, combined two-staged	33.3	5.6	55.6	5.6
Baumann et al. 2009 [4]	2004–2005	6	microsurgical transsphenoidal	66.7	0	16.7	0
De Paiva Neto et al. 2010 [17]	1998–2008	51	endoscopic endonasal	41	0	23.5	0
Sinha and Sharma 2010 [63]	1996–2009	250	microsurgical transsphenoidal, transcranial	31	4.4	76.6	na
Zhao et al. 2010 [75]	1999–2008	44	microsurgical transsphenoidal	61.4	0	na	na
Müslümann et al. 2011 [51]	1994–2008	41	microsurgical transsphenoidal, transcranial	39	2.4	na	na
Guo et al. 2012 [29]	2006–2011	15	transcranial	67	0	33.3	0
Koutourousiou et al. 2013 [41]	2002–2011	54	endoscopic endonasal	20.4	0	25.9	0
Gondim et al. 2014 [26]	1998–2011	50	endoscopic endonasal	38	4	6	na
Wang et al. 2014 [68]	2006–2013	115	endoscopic endonasal	76	0	na	na
Chabot et al. 2015 [12]	2009–2014	6	endoscopic endonasal	50.0	0	na	0
Zhang et al. 2015 [74]	na	112	transcranial, two-staged with transsphenoidal surgery	50.9	2.7	na	7.1
Fu et al. 2016 [23]	2007–2011	21	transcranial	na	0	100	38.1
Kuo et al. 2016 [42]	2002–2009	38	endoscopic endonasal	21.1	0	52.6	2.6
Yildirim et al. 2016 [73]	2009–2014	20	endocopic endonasal	70	na	na	28.6
Han et al. 2017 [30]	2009–2015	62	transsphenoidal, transcranial and combined (simultaneous and two-staged)	35.5	0	40.3	12.9
Karki et al. 2017 [36]	2010–2015	59	microsurgical transsphenoidal	74.6	0	na	3.4
Nishioka et al. 2017 [52]	2008–2015	128	microsurgical transsphenoidal, endoscopic endonasal, simultaneous combined transsphenoidal and transcranial	29.7	0.8	33.6	16.4
Yano et al. 2017 [72]	2002–2015	34	endoscopic endonasal	47.1^a^	2.9	17.6	0
Elshazly et al. 2018 [18]	2008–2016	55	endoscopic endonasal	44	0	25.5	11
Fallah et al. 2019 [21]	2013–2017	35	endoscopic endonasal, transcranial	74.3	na	na	na
Yang et al. 2019 [71]	2012–2015	60	endoscopic endonasal	46.7	na	na	na
Graillon et al. 2020 [27]	2000–2016	19	transcranial, combined two-staged	21	0	36.8	0
Peto et al. 2020 [54]	2015–2018	10	endoscopic endonasal	40	0	na	na
Zheng et al. 2020 [76]	2013–2017	8	endoscopic endonasal	25	na	na	na
Ceylan et al. 2021 [11]	1997–2019	205	endoscopic endonasal	35.1	1.46	6.3	5.85
Chibbaro et al. 2021 [14]	2015–2019	96	endoscopic endonasal	35.4	0	13.5	16.7
Rahimli et al. 2021 [57]	2014–2020	44	endoscopic endonasal	63.6^b^	4.5	22.7	0
Chen et al. 2022 [13]	2015–2021	239	endoscopic endonasal, microsurgical transsphenoidal	19.25	0.84	na	na
Makarenko et al. 2022 [44]	2002–2020	108	endoscopic endonasal, transcranial, combined two-staged	38.9	0.9	24.1	20.0
Micko et al. 2022 [45]	2004–2019	64	endoscopic endonasal, possibly combined with transcranial surgery	28	0	25	na
Ke et al. 2023 [38]	2014–2022	94	endoscopic endonasal, transcranial or combined	63.8	0	18.1	0
Joshi et al. 2024 [35]	2010 −2023	46	endoscopic endonasal, transcranial, combined two-staged	50.0	2.2	na	na
Qiao et al. 2024 [56]	2014–2023	647	endoscopic endonasal, transcranial, simultaneous or combined two-staged	42.0	3.1^c^	na	na
This series	1982–2022	289	transsphenoidal, transcranial, combined two-staged	20.5	2.4	40.1	23.1

^a^Near-total resection (> 90% of initial tumor volume)

^b^Including GTR and near-total resection (90–100% of the tumor)

^c^Including coma

Complication rates for postoperative meningitis and CSF-leaks were 3.5% and 2.8%, respectively, with no significant difference between the surgical approaches. The occurrence of cranial nerve palsy and amaurosis was found to be statistically significant, with the transsphenoidal cohort demonstrating the lowest rates of each (4.0% and 1.5%, respectively). Transcranial procedures involve direct manipulation of sensitive structures such as the optic nerves or cranial nerves close to the base of the brain. This explains the higher risk of damage to these structures.

In general, surgical treatment more often improves than worsens pituitary function in pituitary adenomas [7, 22]. However, this does not apply to giant adenomas, as shown by the present study. The most frequent complication was hypopituitarism, at 34.7%. Overall, no significant difference was found between the surgical modalities and new hypopituitarism. As anterior pituitary insufficiency can be caused not only by surgical treatment, but also by the tumor itself or radiation therapy, the influencing factors must be differentiated. However, a recovery of pituitary function is not to be expected in the majority of patients.

As postoperative hyponatremia can lead to significant morbidity, including neurological deterioration and prolonged hospitalization, sodium levels should be carefully monitored. In this series it occurred in 11.4% of patients and could be controlled in all of them. A total of 27 patients (9.6%) developed diabetes insipidus after the operation, and in about half of these patients it persisted for more than three months after the operation. There was no significant difference in the occurrence of hyponatremia and diabetes insipidus regarding the surgical approaches.

When considering all the different factors influencing tumor resectability and complication rates, it must be noted that this series extended over a period of 40 years. During this time, surgical techniques, endocrinological assays, remission criteria, radiological imaging and data collection methods have all changed.

### Limitations

A major limitation of this study is its retrospective design, which can introduce biases and limitations in terms of data availability and quality. Technological advances have made diagnostic and therapeutic options much more extensive and precise since the earlier phases of the study. Changes in imaging techniques and methods of determining hormone levels could affect the comparability of diagnoses and treatment strategies over the entire period.

## Conclusion

The treatment of GPAs remains a significant challenge in modern medicine. As different characteristics of these large tumors require different approaches, each patient should receive an individual treatment plan. The transsphenoidal approach continues to be the primary consideration for non-prolactinomas. If necessary, it should be combined with a transcranial procedure. Considering both aspects, maximizing tumor resection and minimizing complications, the outcome for patients with GPA has improved dramatically since the early reports of surgical treatment of these tumors [48, 55, 66]. Despite numerous advances, further research and development is needed in terms of minimizing postoperative bleeding, evaluating two-staged or simultaneous procedures as well as the importance of adjuvant therapies.

## Data Availability

Not applicable.

## Abbreviations

CN: Cranial nerve
CSF: Cerebrospinal fluid
GPA: Giant pituitary adenoma
GTR: Gross-total resection
na: Not available
ns: Not significant
RT: Radiotherapy
TC: Transcranial
TS: Transsphenoidal
